# Optimisation Model of Dispersal Simulations on a Dendritic Habitat Network

**DOI:** 10.1038/s41598-019-44716-z

**Published:** 2019-06-03

**Authors:** Henriette Heer, Lucas Streib, Mira Kattwinkel, Ralf B. Schäfer, Stefan Ruzika

**Affiliations:** 1 Institute for Environmental Sciences, Department of Quantitative Landscape Ecology, University Koblenz-Landau, Landau, Germany; 2 Department of Mathematics, University of Kaiserslautern, Kaiserslautern, Germany

**Keywords:** Ecological modelling, Applied mathematics

## Abstract

To predict and mitigate biodiversity loss, a better understanding of species distribution and reliable dispersal models are required. A promising approach in dispersal simulation is the method of spatially explicit graph-based analysis. While graph theory is strongly connected to the field of optimisation in a variety of disciplines, the potential of optimisation has not yet been exploited in dispersal models. We introduce an optimisation model built on a graph-based dispersal simulation of an aquatic invertebrate species with a terrestrial life stage. The model simulates a directed dispersal process and investigates the fastest route to colonise predefined vacant habitat patches. The optimisation model run-time is in general an order of magnitude faster than the underlying simulation and provides the minimum time until the considered habitat patches are colonised under the given landscape structure. These results can then be used to estimate how fast newly formed habitat patches can be reached and colonised. Our model can in principle be adapted to other simulation models and can thus be seen as a pioneer of a new set of models that may support landscape conservation and restoration.

## Introduction

Climate change effects have now been measured throughout all ecosystems and include, but are not limited to, changes in species’ phenology, abundance and distribution^1–3^. Widespread range shifts have been documented with range expansions in warm-adapted species and range contraction in cold-adapted species as well as a consistent trend of northward or westward range expansion of species in the northern hemisphere^1,2,4^. However, some species show little to no net range shifts and range shifts in general remain little understood^5,6^. Range shifts are complex processes driven by population dynamics and dispersal, which themselves are determined by a variety of factors such as changes to the abiotic and biotic environment^7,8^. To understand and mitigate the impacts of climate change on global biodiversity, reliable models of species dispersal are needed^7,9^.

Spatially explicit dispersal models for freshwater insects are scarce^10^. This scarcity is primarily due to the lack of data, as field studies are very costly and methodical limitations complicate the reliable derivation of dispersal distances for (freshwater) insects^11,12^. A deeper knowledge in the field of species distribution and therefore species dispersal — as one of its key factors — is required to allow for prediction of the effects of climate change^13^.

The method of spatially explicit graph-based analysis is one of the most promising approaches to model dispersal of aquatic individuals^10,14^. This was adopted from the field of graph theory and gained popularity in landscape ecology and conservation biology in recent years^15,16^. While spatial graphs have become an important tool in terrestrial landscape ecology, they are still rarely used in aquatic ecosystem modelling^17^. The advantages of this graph-based structure are numerous. First, graphs are particularly flexible as vertices can represent multiple ecologicial properties, e.g. single individuals, whole populations or, as most appropriate in dispersal models, habitat patches. Second, vertices are connected by links which specify the connectivity relationship^15,16,18^. Furthermore, spatially explicit data derived from geographic information systems (GIS) can be combined with information on dispersal characteristics of the considered species. At the same time only relatively few data are required^17,19^.

Although graph-based structures are now commonly used in ecological models such as dispersal simulations, the potential of optimisation on graphs has — to the best of our knowledge — not yet been exploited in dispersal models. Mathematical optimisation and graph theory are strongly connected in various other disciplines as finance, logistics, engineering and transportation and optimisation is used ubiquitously to solve a variety of problems in various disciplines^20,21^. In general, optimisation approaches are used to identify the ‘best’ solution for a given problem. In the context of dispersal simulations, optimisation can be used to find the fastest way for a species to disperse and related to this the minimum time required to colonise a habitat. Optimisation involves modelling a directed dispersal in contrast to the undirected dispersal that is usually simulated. This approach needs less information on dispersal strategies that define which habitats are preferably colonised and how to divide the dispersing biomass between all neighbouring habitat patches. This is an exceptional advantage, as collecting data is costly and making assumptions is error-prone. On the other hand, an optimal solution only determines bounds for a given problem and provides thus a more general and less specific solution to given research questions such as how long it takes a species to colonise a habitat or which habitats are colonised first.

This study applies tools from optimisation on graphs to a simulation model for dispersal. Given a graph-based model to simulate the spread of a generic aquatic invertebrate with a terrestrial life stage, an optimisation model is derived as a surrogate for the former model. It yields lower bounds on the colonisation time of specific habitats, which provide the minimum time until the considered habitat patches are colonised under the given landscape structure. These results can be of great value, as they identify how far the considered species can disperse within a given time frame and thus give an indication of maximum possible range shifts. At the same time, the model can be used to estimate how fast newly formed habitat patches can be reached and colonised. This information can then be used to modify the underlying connectivity to make habitats more accessible or to study the impact of land use changes. Although being specific for this simulation model, the general idea of deriving a surrogate can in principle be adapted to other simulation models.

Our optimisation approach differs vastly from the least cost path method^22^. The least cost path technique indentifies a shortest connection between a pair of nodes, but does not consider the interaction of multiple source habitats. Our model also takes the possibility into account, that a habitat patch can be reached by more than just one neighbouring patch at a time and thus many patches can jointly colonise a habitat patch. Circuit theory^23^ on the other hand incorporates the possibility of multiple pathways between habitat patches. In contrast to our model, however, it is largely applied to random walk theory. While it can be used to obtain an estimate of dispersal time, it is not designed to calculate lower bounds for these – the main feature of our optimisation model.

The interaction between optimisation and simulation is not a new field of study. However, note that our approach significantly differs from the topic of “simulation optimisation” (SO)^24^. SO is an umbrella term for techniques that search for specific settings of the input parameters to optimise stochastic simulations and often only depend on input - output data from these simulations. In contrast, our model is based on a deterministic simulation and can be classified as a traditional mathematical optimisation technique. Furthermore, we modify well-known mathematical optimisation techniques to develop a model as a surrogate of an ecological simulation model that answers different, but related questions to the simulation model.

We first present the graph-based simulation model for the distribution of a generic aquatic invertebrate with a terrestrial life stage. From this simulation model, we then derive a mathematical optimisation model in form of a mixed integer programming model^25–27^. This model modifies and utilises the concept of dynamic network flows^28,29^. Network flows are typically applied in transportation systems, air traffic control, production systems and financial flows^30–32^ but have not yet been used in ecology. Given some vacant habitats as targets, the optimisation model finds a route to colonise those habitats as quickly as possible.

## Methods

### Simulation model

We developed a dynamic, spatially explicit dispersal model for a generic aquatic invertebrate species with a terrestrial life stage. The simulation model can also be adapted to vertebrates with both aquatic and terrestrial life stages^33,34^. The simulation is based on a habitat network embedded in an artificial landscape defined by four land cover classes (Table 1).

**Table 1:** Dispersal costs and ratio per land cover class. The percentages refer to the underlying Neutral Landscape Model (NLM) that was used to create the landscape (Suppl. Inf. S1). A real stream network on a finer scale is added to the landscape and cells intersecting with the river network are declared as ‘aquatic’.

Class name	Percentage	Dispersal costs
agriculture	66.6%	50.0
forest	11.1%	75.0
urban	22.2%	100.0
aquatic	—	25.0

**Figure 1:**
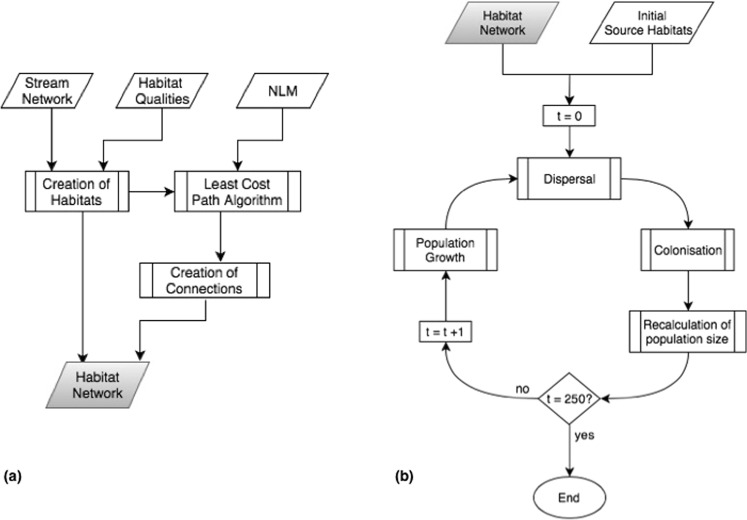
Flowchart of the simulation model. A detailed description of each subprocess (double rectangles) can be found in Supplementary Information S1. (**a**) Creation of habitat network, which is then used as input for the dispersal simulation depicted in (**b**).

Since the overland dispersal of invertebrates during their terrestrial life stage is influenced by land cover (e.g. preference for specific land cover classes), we assigned dispersal-related costs to these land cover classes^35^. These costs determine the spatial connectivity between habitats. They were chosen to represent landscape permeability with a relatively energy efficient dispersal through aquatic and open agricultural terrain, whereas forests and urban areas represent a rather costly dispersal path. Habitats are located along a stream network that is embedded in the landscape and are assigned with random habitat qualities which determine the maximum population that can be sustained in a habitat patch, called carrying capacity. Some of the habitat patches are randomly chosen as initial source habitats and considered colonised at the start of a simulation. The dispersal process from those patches is modeled as a dynamic process using a modified individual based model (Suppl. Inf. S1). The simulation is based on the demography-related processes population-growth (depending on habitat quality) and density-dependent emigration^36–38^ (Fig. 1). Consequently, the amount of dispersing biomass primarily depends on population size and habitat quality as controlling factors of maximum population size (carrying capacity)^39,40^. In a colonised habitat patch, the population initially grows exclusively due to immigrating biomass from neighbouring source habitats. After a predefined threshold of biomass is reached, it turns into a source habitat and an additional population growth as well as emigration is simulated (Fig. 1). We assume that habitat patches that can be reached at low dispersal costs are preferably colonised^41^ and thus receive a bigger share of biomass, where the dispersal costs depend on both the distance to a source habitat and the land cover classes traversed (Suppl. Inf. S1).

The maximum dispersal distance was set to 2500 m through open agricultural land^42,43^. Consequently, on a cost raster with a cell-size of 100 m × 100 m, our model species was assigned a maximum budget of 1250 cost units (‘agriculture’ 50 cost-units · 25 raster cells) and two habitat patches were considered connected, if the dispersal costs between them was less than *C*_max_ = 1250. This results in a graph-based habitat network *G* = (*V*, *E*), where the set of vertices *V* subsumes all habitat patches and the edge set *E* contains all connections between them (Suppl. Inf. S1). The same habitat network is used as basis for the optimisation model.

One habitat network was created as basis for all following simulations. 50 sets of initial source habitats were randomly selected as simulation input (see *Initialisation*). As the simulation is deterministic, a single simulation per model input was sufficient. Although the design of a habitat network has a strong influence on species dispersal, we considered only one habitat network, as its influence was beyond the focus of this study.

### Optimisation model

#### Process overview

The simulation model assumes that close habitats are preferably colonised and applies a colonisation route accordingly (Suppl. Inf. S1). Here, a colonisation route is a detailed plan of the species’s movement in the network over time, which leads to a colonisation success. In terms of the time expanded network (see next subsection), we define a colonisation route as a set of paths connecting the source to the corresponding copies of the destination habitats in the time expanded network combined with the information about the exact amount of biomass that is traveling along that path. Since this assumption has a strong impact on the colonisation time of vacant habitats, we design an optimisation model that identifies a route to colonise specific, predetermined habitat patches as quickly as possible. Analogously to the simulation model, a habitat network is created and a predefined share of habitat patches are randomly selected as initial source habitats. Additionally, a set of habitat patches is selected as destination habitats. The fully colonised initial source habitats initiate the dispersal process and dispersal is directed towards the selected destination habitats in contrast to the undirected dispersal in the simulation model. The model output is a provable lower bound on the colonisation time of the simulation model and guarantees that the predefined destination habitats will not be colonised earlier — independent of the dispersal route. Furthermore, the run-time of the optimisation model is substantially faster than the simulation model. The optimisation model is instantiated on the same landscape model as the simulation. The graph-based habitat network *G* = (*V*, *E*) created by the simulation model (Suppl. Inf. S1) was used as a representation of the investigated area. Similar to the simulation model, population growth is not taking place before a specific threshold *T*_*SH*_ is reached. *T*_*SH*_ is the minimum viable population — a simplified threshold that specifies the smallest amount of biomass needed for a species to persist in a habitat patch. However, to simplify the model, once the threshold is reached, the population will grow to the habitat specific carrying capacity *K*(*v*) (Suppl. Inf. S1) within one time step. Thus, a habitat with a population size of at least *T*_*SH*_ units of biomass is considered to be fully occupied and a source habitat in the following time step. After a loss of biomass due to dispersal, the population of a habitat patch is set again to the carrying capacity in the same time step. To sum up, each source habitat *v* has a constant population size of *K*(*v*) and can release an additional amount of up to *S*_*DIS*_*K*(*v*) biomass during the dispersal process.

The model utilises the method of time expanded networks^28,29^ and solves a mixed integer program (MIP)^25–27^ to compute the desired bounds.

#### Habitat network

**Figure 2:**
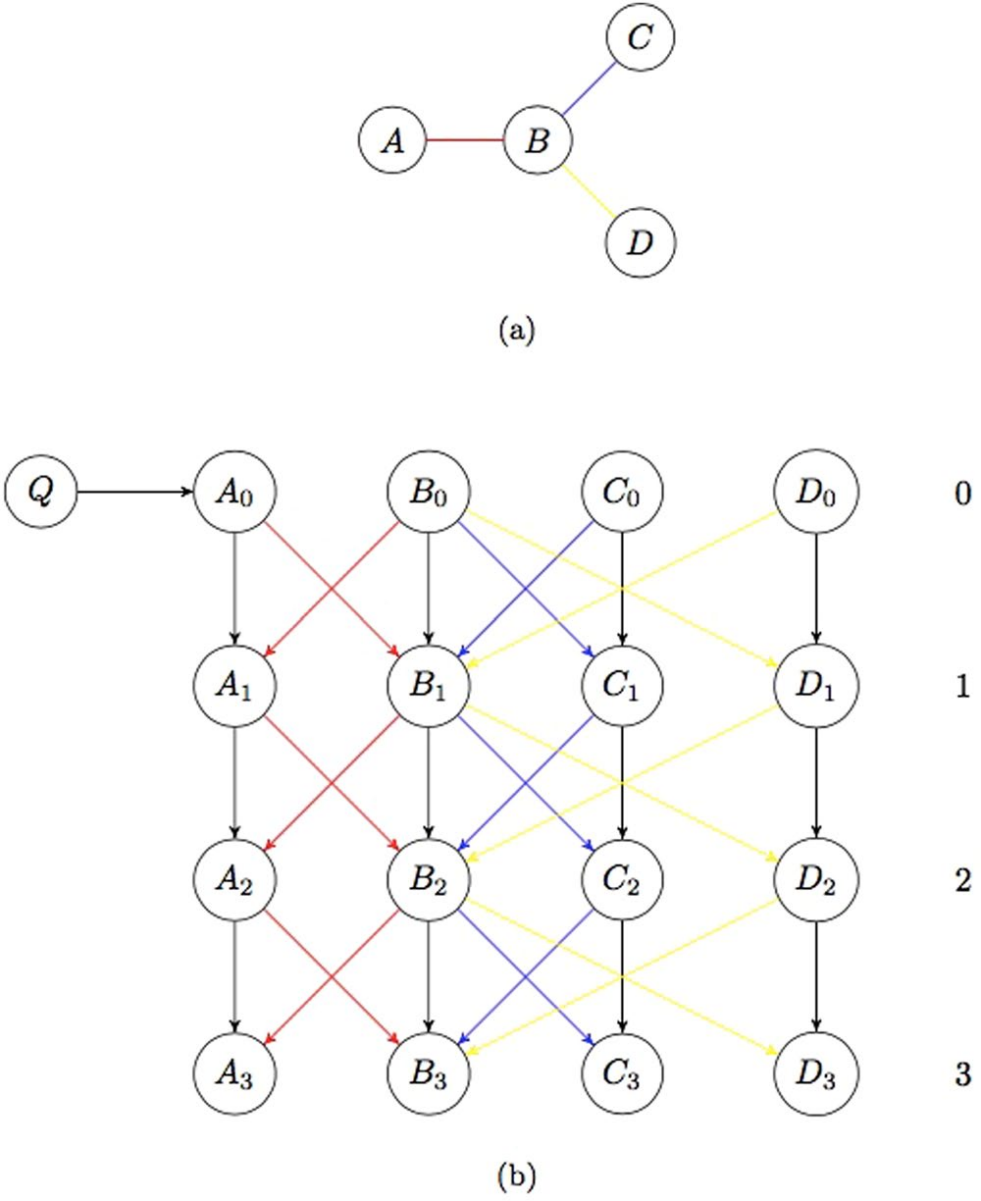
(**a**) Habitat network (**b**) Corresponding time expanded network with time horizon 3 and source habitat *A*. Connections in (**b**) representing a specific connection in (**a**) use the same colour. Black connections in (**b**) are artificial connections indicating remaining in the same habitat patch from one time step to the next.

A time expanded network *G*_TEN_ = (*V*_TEN_, *E*_TEN_) (Fig. 2) is created to represent the graph-based habitat network *G* = (*V*, *E*) and to store the population size of each habitat patch in every time step. A time expanded network is a directed network (i.e. connections between vertices have a direction and can only be traversed along this direction)^20^ with one copy of each habitat patch of the underlying habitat network per time step (*time layer*) and connections between habitat patches in consecutive layers.

Let *T* be the time horizon, i.e. the maximum number of time steps considered in the model. For each habitat patch *v* ∈ *V*, *T* + 1 copies *v*_0_, …, *v*_*T*_ are constructed which represent the habitat patch *v* at time steps 0, …, *T*. For each connection (*u*, *v*) ∈ *E* between two habitat patches *u* and *v* and each time step *t* = 0, …, *T* − 1, two directed connections (*u*_*t*_, *v*_*t*+1_) and (*v*_*t*_, *u*_*t*+1_) are introduced in the time expanded network. To model the possibility of remaining within a vertex between two consecutive time steps, connections (*v*_*t*_, *v*_*t*+1_) are introduced for all *t* = 0, …, *T* − 1 and for all habitat patches *v* ∈ *V*. As is typically done in time expanded networks^28,29^, a super source *Q* is introduced together with connections (*Q*, *q*_0_) to the first copy of each initial source habitat *q*.

All habitat patches and connections are equipped with the original data: each copy of a habitat patch *v* ∈ *V* is assigned the same dispersal capacity value as the original, *u*(*v*_*t*_) := *S*_*DIS*_*K*(*v*). Each copy of a connection is assigned the same dispersal costs as the original ones, while the artificial connections (connections between copies of the same habitat patch and all connections from *Q*) are assigned zero cost (Fig. 2).

#### Mixed integer program

In the following, an optimisation problem is formulated which yields the minimal colonisation time as described above. To this end, techniques from integer programming are applied and the result is a so-called mixed integer programming problem (MIP)^25–27^ which will then be solved by some integer programming solver.

Binary decision variables *x*(*v*_*t*_) are introduced for each habitat *v* ∈ *V* and each time step *t*. If *x*(*v*_*t*_) = 1, then *v*_*t*_ is a source habitat and otherwise it is not. Furthermore, for each connection *e* ∈ *E*_TEN_ in the time expanded network, a real variable *f*(*e*) ≥ 0 specifies the amount of biomass traveling along this connection. Moreover, binary variables *x*(*t*) are introduced for each time layer *t* ∈ {0, …, *T*} indicating if all destination habitats in the corresponding time layer are source habitats. In the following, *δ*^+^(*v*) denotes the set of all connections leaving *v* and, analogously, *δ*^−^(*v*) specifies the set of incoming connections into *v*.

The objective function of the MIP minimises the sum of all time layer decision variables multiplied by *t*, over all *t*.

$${\rm{\min }}\,\sum _{t=0}^{T}\,tx(t)$$

Here, the cost coefficient *t* with which the decision variable is weighted, corresponds to the time and thus increases over time. Thus, in view of the minimisation objective, it is desirable, to send biomass to the destination habitats as quickly as possible. This objective function was adopted from models for the so-called quickest flow problem and the earliest arrival flow problem and guarantees that the fastest way to colonise the specific destination habitats will be found^44^.

The first set of constraints

1 $$\begin{array}{ll}{T}_{SH}x(v)\le \sum _{e\in {\delta }^{-}(v)}f(e)\, & \forall v\in {V}_{{\rm{TEN}}}\backslash \{Q\}\end{array}$$

ensures that a habitat *v* at time step *t* can only become a source habitat, if the incoming amount of biomass at time step *t* plus the biomass from the previous time step (represented as *f*((*v*_*t*−1_, *v*_*t*_))) are at least *T*_*SH*_.

The second set of constraints

2 $$\begin{array}{ll}f({v}_{t-1},{v}_{t})=\sum _{e\in {\delta }^{-}({v}_{t-1})}f(e)\, & \forall v\in V,t\in \{0,\ldots ,T\}\end{array}$$

sends all biomass of a habitat patch from the previous time step *t* − 1 to the current time step *t*.

The constraints

3 $$\begin{array}{ll}\sum _{e\in {\delta }^{+}(v)}\frac{f(e)}{1-C(e)\frac{1}{{C}_{{\rm{\max }}}}}\le u(v)x(v)+\sum _{e\in {\delta }^{-}(v)}f(e)\, & \forall v\in {V}_{{\rm{TEN}}}\end{array}$$

are the crucial constraints of the model. They ensure that a source habitat does not emit more than an upper limit of biomass and simultaneously take into account that only a fraction of the biomass emitted reaches the connected habitats. On the left hand side of the inequality, the emitted biomass *f*(*e*) is reduced by the mortality rate $C(e)\frac{1}{{C}_{{\rm{\max }}}}$, where *C*(*e*) represents the dispersal cost of a connection *e* and *C*_max_ is the maximum dispersal cost (see simulation model). This reduction represents the mortality of dispersing biomass. The higher the dispersal costs *C*(*e*) of a connection *e*, the smaller the share of biomass traversing connection *e* to reach the destination. The right hand side now ensures that a source habitat *v* does not emit more than *u*(*v*) units of biomass. If *v* is no source habitat, then *x*(*v*) = 0 and no additional biomass can be emitted. The additional amount ${\sum }_{e\in {\delta }^{-}({v}_{t})}f(e)$ is the amount of biomass that stays in the habitat (constraint 2) and is sent into the next time step.

The constraints

4 $$\begin{array}{ll}f(Q,{q}_{0})={T}_{SH}\, & \forall q\in {H}_{{\rm{Start}}}\end{array}$$

ensure that all initial source habitats *q* ∈ *H*_Start_ are fully colonised (according to *T*_*SH*_) at time step 0, where *H*_Start_ is the set of all initial source habitats.

The fifth set of constraints

5 $$\begin{array}{ll}|{H}_{{\rm{dest}}}|x(t)\le \sum _{s\in {H}_{{\rm{dest}}}}\,x({s}_{t})\, & \forall t\in \{0,\ldots ,T\}\end{array}$$

ensures that the time layer variable *x*(*t*) can only be set to one, if all destination habitats are colonised in that time layer, where *H*_dest_ is the set of destination habitats, and the constraint

6 $$\sum _{t=0}^{T}x(t)\ge 1$$

requires that all destination habitats have to become source habitats eventually.

All in all, the following MIP is obtained, which can now be solved with the help of any MIP solver such as the one provided by Gurobi^45^.

$${\rm{\min }}\,\sum _{t=0}^{T}tx(t)$$

subject to (1)–(6)

$$\begin{array}{ll}f(e)\in {{\mathbb{R}}}_{+} & \forall e\in {E}_{{\rm{TEN}}}\\ x(v)\in \{0,1\} & \forall v\in {V}_{{\rm{TEN}}}\\ x(t)\in \{0,1\} & \forall t\in \{0,\ldots ,T\}\end{array}$$

#### Time horizon

The choice of the time horizon is crucial to the model performance. Since the calculations are executed on a time expanded network, the model input is linear in *T* and thus a large time horizon will lead to an exorbitant model run-time, while a time horizon chosen too small will not return any information as the MIP will turn out to be infeasible. Thus, a good approximation of the maximum number needed will vastly improve the model performance. The following procedure was used to find the appropriate time horizon for a given habitat network and its specific initial source habitats and destination habitat.

With the help of the Python module ‘Networkx’^46^ and taking the dispersal costs into account, a shortest path was calculated from each initial source habitat to the destination habitat. Based on these results, the nearest initial source habitat was identified and the destination habitat was colonised with successively colonising the habitat patches *v*_*i*_ from the nearest initial source habitat along the shortest path *P* = (*v*_1_, …, *v*_*k*_) to the destination habitat, using the colonisation rules of the optimisation model. This can be calculated with the following formula:

$$T{H}_{1}=\sum _{i=1}^{n}\lceil \frac{{T}_{SH}}{K({v}_{i}){S}_{DIS}(1-\frac{C({v}_{i},{v}_{i+1})}{{C}_{{\rm{\max }}}})}\rceil ,$$

Since this is only one of many feasible possibilities to colonise the specific destination habitat, the minimum of all possibilities is clearly smaller. To obtain an even closer bound, the same procedure was performed with the second nearest initial source habitat, if available, obtaining a second bound *TH*_2_ for the time horizon. Although the cumulative dispersal costs from the second initial source habitat to the destination habitat is not smaller than from the first one, the second bound can be smaller than the first one due to rounding to integers in the formula, for instance. Thus, the minimum of both bounds is taken as the time horizon. In some cases both bounds *TH*_1_ and *TH*_2_ turned out to be too big and thus the minimum of both bounds and 30 was selected as time horizon for all model runs:

$$T=\,{\rm{\min }}\,\{T{H}_{1},T{H}_{2},30\}$$

If the MIP with this time horizon was infeasible, a new time horizon was set to be the minimum of *TH*_1_, *TH*_2_ and 60 and the procedure was repeated with higher multiples of 30 if necessary. Although this led to a slower performance for model runs with an outcome bigger than 30 (due to solving a smaller, infeasible model and repeating the process), this method was used as it yielded a speedup for the majority of all model runs. Indeed only one percent of all model initialisations needed a time horizon bigger than 30.

### Initialisation

The habitat network constructed by the simulation model was used for both models. To compare the optimisation model with the underlying simulation, one habitat network was chosen to represent the underlying landscape structure and both models were instantiated with the same model parameters (Suppl. Inf. Table S2). For each simulation model run a new set of initial source habitats was chosen. The same set was taken as optimisation model input for multiple model runs. Additionally a set of destination habitats was randomly chosen and each of the destination habitats was combined individually with each set of initial source habitats as input for an optimisation model run. As dispersal is undirected in the simulation model and the simulation model is deterministic, one model run per set of initial source habitats was sufficient to investigate the colonisation time of all possible habitat patches. For the optimisation model, dispersal is directed and different destination habitats have to be considered individually.

The considered extent of 50 km × 50 km of the stream network accounts for a total of 19,490 pixels classified as ‘aquatic’. As described in the simulation model (Suppl. Inf. S1), a random selection of 10% of these pixels were chosen as habitat patches. Together with the connections created by the least cost path algorithm (Suppl. Inf. S1), these habitat patches form the habitat network. One habitat network was created and then used for all model runs. For each simulation model run, 10% of those habitat patches were randomly selected as initial source habitats. In total, 50 distinct sets of initial source habitats were chosen and taken as model input of the simulation model. Additionally, 50 habitat patches were elected as destination habitats and each set of initial source habitats combined with each destination habitat individually were taken as model input for the optimisation model. Although the optimisation model was developed to determine the minimum colonisation time for a set of (multiple) destination habitats, we focus on a single destination habitat from here on. This makes it easier to compare outcome and run-time of the two models, as the combination of different destination habitats has a strong influence on them. Both models were implemented in Python 2.7. The MIP solver provided by Gurobi^45^ was used to solve the optimisation problem. Both models were executed on a server with the Ubuntu release 16.04.3 LTS, Intel Xeon 16 core processor 2.50 GHz with memory of 31.4 GB and timed with the help of the Python module ‘Timeit’.

### Analysis of models

First, we compare the optimisation model outcome with the model input by examining the dispersal distance of all initial source habitats from the specific destination habitat for all optimisation model runs. Second, to compare the outcome of both models, we investigate the number of time steps to colonise the considered destination habitats and compare the outcome of each of the 2,500 optimisation model runs to the time step in which the corresponding destination habitat changed its status into a source habitat in the simulation model for the first time (Suppl. Inf. S1). Third, we compare the run time of both models.

## Results and Discussion

### Analysis of model results

**Figure 3:**
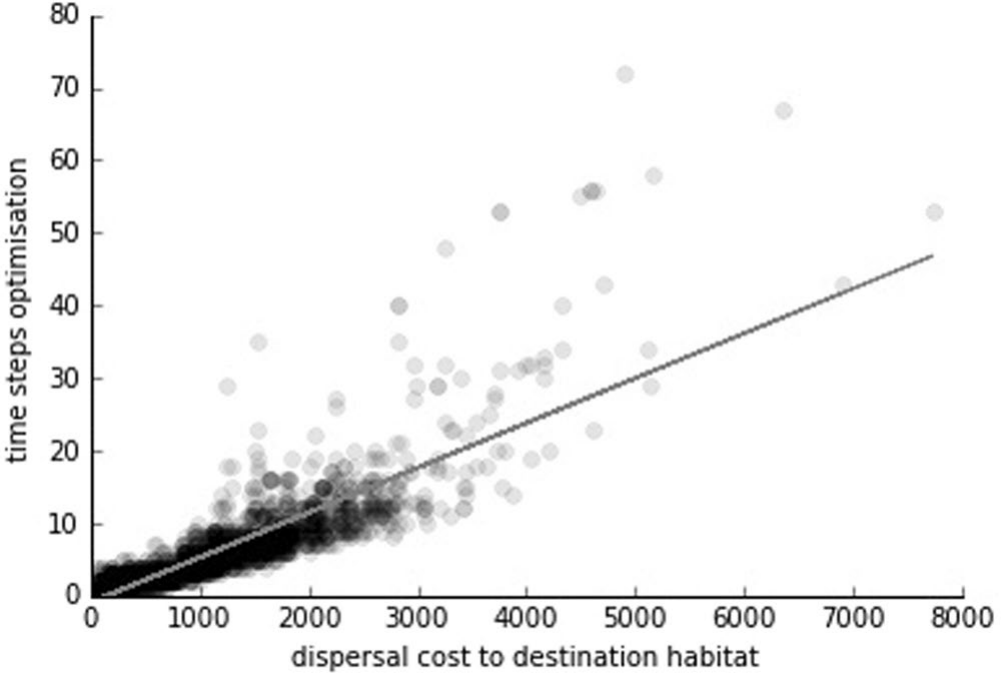
Relationship between the time steps found by the optimisation model and the cumulative dispersal cost from the nearest (i.e. most cost efficient) connected initial source habitat to the destination habitat of the corresponding model run. Each black mark in the plot corresponds to one of the 2,500 model runs. The grey line is the linear regression.

We found a positive correlation (r = 0.85) between the distance (in terms of dispersal costs) from the nearest initial source habitat to the destination habitat and the minimum colonisation time (in terms of time steps) calculated by the optimisation model (Fig. 3).

By contrast, the average dispersal costs from all initial source habitats (that are connected to the destination habitat) to the destination habitat is only weakly correlated with the optimisation model outcome (r = 0.34). This fact indicates that not all initial source habitats play an equally important role in the colonisation of the corresponding destination habitat. In fact, the dispersal costs of close initial source habitats have a much stronger influence on the optimisation model outcome. To conclude, the optimisation model colonises those habitat patches faster, that are more cost-efficient to reach. This was not implemented in the optimisation model and conforms to common literature and the assumption made for the simulation model that close habitat patches are preferably colonised^41,47^. Thus, the optimisation model responds in a similar fashion to the simulation model and observations in common literature, which can be seen as a partial validation of the optimisation model.

In a second step, the outcome of both models was compared. In less than 2% of the model inputs (41 out of 2,500), the destination habitat in the simulation model was not colonised after 250 time steps. These model instances were omitted in this analysis, as the simulation model outcome is unknown. However, they also had a considerable large optimisation model outcome with a mean of 32.4 time steps (range from 2 to 72, median 29).

**Figure 4:**
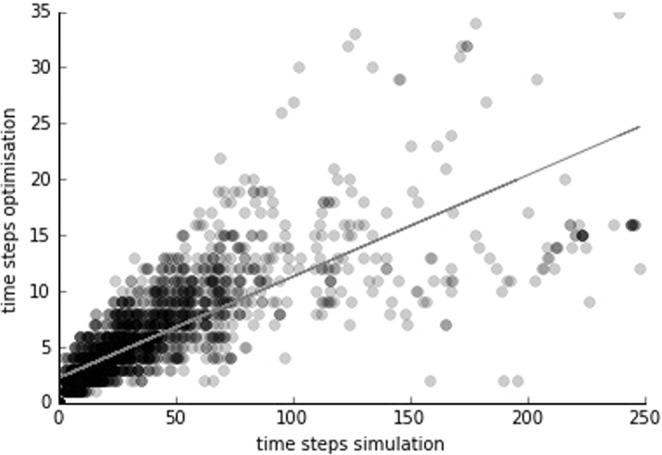
Correlation between time steps needed for colonisation of destination habitat of simulation and optimisation. Each black mark corresponds to a single run of the optimisation model and the corresponding result from the simulation. The grey line is the linear regression.

The optimisation model colonises a destination habitat on average 6.8 times faster than the simulation model (Fig. 4). Thus, the optimisation model not only gives lower bounds on the colonisation time of the simulation model, but also gives an estimate of the expected outcome of the simulation. However, this estimate is subject to considerable uncertainty and ranges from 1 to 98-fold for different model runs. Model runs with the highest deviation from this average have an optimisation model outcome of 2 time steps (Fig. 4). These high deviations happen particularly in dense areas. In the simulation model, the initial source habitats will have many neighbouring habitat patches and dispersing biomass is distributed among many neighbours (Suppl. Inf. S1) — leaving only a small share for the designated destination habitat. The optimisation model on the other hand sends all available biomass directly towards the designated destination habitat. Accordingly, the destination habitat will be colonised much faster in the optimisation model compared to the simulation.

### Model run times

The mean time of a simulation model run is 318 seconds with a standard deviation of only 5 seconds. The optimisation model was much faster on average, but also included some model runs with larger run-times. A run of the optimisation model takes 17 seconds on average and is thus almost 20 times faster than the corresponding simulation model. However, the performance varies vastly for different settings. The 90th percentile is 26 seconds and the 50th percentile is 3.5 seconds, while 16 of all 2,500 optimisation model runs (0.64%) were slower than the simulation model. The computationally most expensive model instances also have a rather large model outcome. This may be due to the way the time horizon was chosen and the fact that a higher time horizon was needed. Similar to a single destination habitat, the run time of the optimisation model with multiple destination habitats varies depending on the input. However, the run time takes on average 60 seconds and is thus considerably slower than the single destination case. On the other hand, this is still roughly 5 times faster than the simulation model run time. Thus, the run time advantage also holds for multiple destination habitats.

To conclude, the optimisation model is one order of magnitude faster than the simulation model. On the other hand, some model instances are hard to solve and less than one percent of the instances needed more time than the corresponding simulation model.

It is important to point out that the two models pursue different goals and are thus difficult to compare. While the simulation model investigates, inter alia, the distribution of colonised habitat patches after a given number of time steps, the optimisation model examines the minimum number of time steps needed to reach a specific habitat patch. Thus our model is not a mere surrogate which answers the same questions with less accuracy, but provides results that cannot be found using the original model — in contrast to other surrogate models like (Gaussian process) emulators. Gaussian process emulators are statistical models that approximate unknown output of a complex and time-consuming simulation. Given some design data consisting of input - output pairs, the simulation output of further inputs are approximated by a Gaussian process^48^. Emulators are orders of magnitude faster than their original model^49,50^. Thus, considering the performance gains, the optimisation model can compete with emulators, but would be considered a slow speed-up.

On the other hand, the simulation model run-time strongly depends on the total number of simulated time steps. The total number of 250 time steps was chosen such that most habitat patches were reachable within that time frame and such that the number of time steps was not so high that run-time was needlessly increased. A better run-time comparison could be achieved by adjusting the fixed number of 250 time steps to an input-dependent number (for example by stopping the simulation when the destination habitat in focus is colonised). This adaption, however, changes the focus of the simulation model and is not intended.

### Example

**Figure 5:**
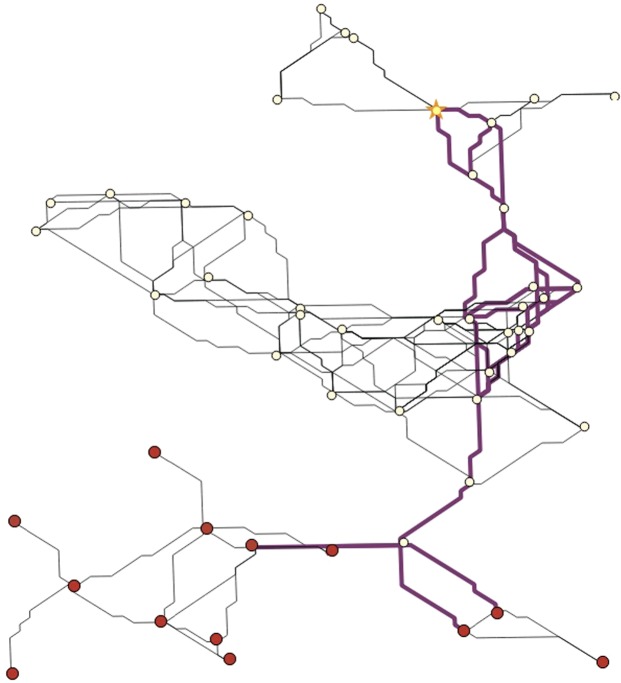
Application of optimisation model on a habitat network. The red nodes represent initially colonised habitat patches, the yellow star is the corresponding (uncolonised) destination habitat. The thick, purple lines show the dispersal paths displayed by the optimisation model.

In this section we demonstrate how our optimisation model can be applied in landscape management. Figure 5 shows an artifical landscape (created as described in the Methods section) where a species is present in the southern area of the landscape (initially colonised habitat patches are represented by red circles). Due to climate change, more patches located in the northern part of the landscape become habitable. To evaluate how to facilitate the spread of our focal species to the newly formed habitat patches, a central patch is chosen as destination habitat (yellow star) as model input.

With the current underlying landscape scenario, the destination habitat is only colonised after 34 years, if the species disperses along the given paths. This result can then be used by landscape and freshwater managers to facilitate the colonisation by strengthening the connections as well as the habitat patches along them to allow for faster and easier traversal.

One should keep in mind, however, that the focus of this model is to determine a minimum colonisation time rather than identifying suitable areas in the landscape that yield the largest improvement (in terms of colonising the destination patch as fast as possible) if enhanced.

### Challenges and outlook

To construct the optimisation model from the simulation, the habitat network was transformed into a time expanded network. Additional decision variables were introduced to memorize the fully populated habitat patches. The time expansion results in an exponentially bigger input size, implying a loss of computational efficiency. At the same time, this is a common structure to monitor changes (here: of population size in habitat patches) over time^30–32^. Furthermore, this structure can later be exploited to integrate changes over time, for example in habitat quality, into the model.

Translating the dispersal process and population growth of the simulation model to linear constraints is the most challenging part in creating the optimisation model. In particular, integrating a more realistic population growth process into the optimisation model would increase the complexity considerably, as it demands additional decision variables and constraints. These were omitted in our optimisation model and the simulation of population growth was simplyfied to ensure a faster and simpler model. On the other hand, if the species in focus has very slow or complicated population dynamics, this simplification may lead to a huge underestimation of the colonisation time. Linear constraints are the core of linear programming and the main challenge in adapting the optimisation model to other simulation models will be to translate complex processes into linear equations.

Some studies also found inverse density dependent dispersal patterns for damselflies^51^. Inverse density dependent dispersal characterises the pattern that occurs when individuals from sparsely populated habitat patches gravitate towards more densely populated patches. In the current study, we focus on dispersal from colonised to empty patches, not between two colonised ones. Therefore, such inverse densitiy-dependet dispersal is not relevant for our research question. Furthermore, the survival probability of a small population (i.e. a small amount of biomass in our study) reaching these patches and dispersing even further to uncolonised habitat patches is negligible. Thus, both the simulation and the optimisation model focus on dispersal that occurs at the carrying capacity threshold. However, both models can be adapted to different dispersal patterns.

A detailed analysis of the model outcome can lead to a better understanding of range shifts. For example, the lower bounds found by the optimisation model can be used to identify important habitat patches for species dispersal and to evaluate the strength of the connection between certain habitat patches and their surroundings. This is especially interesting, as connectivity is a major concern for population survival and reduction of extinction risk^52,53^. The optimisation model allows to make decisions where and how to conserve habitat patches or landscape sections to secure a better habitat connectivity. At the same time, the model can be used to identify the optimal case to (re-)colonise habitat patches that arose or recovered due to climate change or other effects.

The model can readily be adapted to other dispersal simulation models and can thus be seen as a pioneer of a new set of models with a variety of applications such as dispersal prediction and habitat conservation and restoration.

## Supplementary information

Optimisation Model of Dispersal Simulations on a Dendritic Habitat Network - Supplementary Information
