# Supplementary material for: Optimisation Model of Dispersal Simulations on a Dendritic Habitat Network
Source: Sci Rep. 2019 Jun 3;9:8202. doi: 10.1038/s41598-019-44716-z (PMC6547651; doi:10.1038/s41598-019-44716-z)
Supplement: Supplementary file 1 — Optimisation Model of Dispersal Simulations on a Dendritic Habitat Network - Supplementary Information [file 41598_2019_44716_MOESM1_ESM.pdf]

Optimisation Model of Dispersal Simulations on a Dendritic Habitat Network

Henriette Heer, Lucas Streib, Mira Kattwinkel, Ralf B. Schäfer, Stefan Ruzika

1 Supplementary Methods

Simulation Model

Habitat Network

An artificial landscape raster was created with an extent of 50 km × 50 km and a resolution of 25 m × 25 m per pixel. The Neutral Landscape Model algorithm 'simple random'[1] was used to produce a landscape defined by four land cover classes with varying shares and class related dispersal cost values per pixel.

A section of the dendritic stream network for the German federal state Rhineland-Palatinate[2] was used to include a realistic arrangement of streams into the generic landscape to obtain a more realistic distribution of aquatic cells. Thereto, each raster-cell intersecting a stream segment is assigned with a comparatively low dispersal cost value (25.0) and classified as 'aquatic'. The resulting landscape is then defined by four classes (Tab. 1).

Table 1. Dispersal costs and distribution per land cover class. The percentages refer to the underlying Neutral Landscape Model (NLM) that was used to create the landscape. A real stream network on a finer scale is added to the landscape and cells intersecting with the river network are declared as 'aquatic'.

class name	percentage	dispersal costs
agriculture	66.6%	50.0
forest	11.1 %	75.0
urban	22.2%	100.0
aquatic	-	25.0

Finally, the raster is resampled to a pixel-size of 100 m × 100 m to create a landscape in which mutual interferences between segments with different classes are considered. The GIS algorithm 'gdalwarp' with the method 'average' is applied for resampling[3], which computes the average of all contributing pixels (here 16 pixels of size 25 m × 25 m).

We assumed a heterogeneous landscape structure and thus a wide range of habitat qualities has to be considered.[4] Therefore, a specific habitat quality HQ (in a predefined range) is randomly assigned to a predefined share S_{HP} of all 'aquatic' pixels at the start of the simulation. The remaining pixels were assumed to have a low habitat quality and were not considered habitat patches. Sutherland & Norris[5] indicate that the habitat quality is strongly correlated to demography. Hence, a low quality results in a reduction of fitness. Accordingly, an individual capacity limit K per habitat patch is deduced from these habitat qualities:

$$K(v) = 100.0 \cdot HQ(v).$$

The factor 100.0 was chosen as literature sources provide maximal abundance ranges from 68.3 to 211.5 per 100 m.[6,7].

Since the generic model species has a terrestrial life stage, the dispersal is not restricted to the stream network. For this reason, all possible connections between patches with an euclidean distance less or equal to the expected maximum dispersal distance are considered for the construction of the habitat network. To determine dispersal costs between connected habitat patches in the network, the connections as well as the cost-raster serve as input for a least cost path analysis. Thereto, the function route_through_array from the skimage.graph package[8] for the programming language Python is used. This algorithm computes specific least cost paths from each habitat patch to all connected habitat patches on the cost-raster. Moreover, the corresponding minimum cumulative dispersal costs per connection are calculated and assigned as dispersal costs to the specific connection.[9] Connections with costs exceeding a maximum threshold, are not considered.

The result is a graph-based network $G = (V, E)$ consisting of the set V of all habitat patches including their habitat qualities and the set E of all connections between two different habitat patches and their specific dispersal costs.

Dispersal Simulation

At the start of each dispersal simulation, an initial model state is defined by a random selection of a predefined share S_{SH_i} of all habitat patches as initial source habitat patches. These habitats are fully colonised according to their specific capacity limit and serve as initial source habitats for the colonisation of the remaining, not yet populated habitat patches.

In each of the subsequent time-steps, population-growth is simulated for all source habitats and dispersal takes place, where a share S_{DIS} of the habitat's population is split amongst the neighboring habitat patches depending on their dispersal costs. As soon as a species-specific threshold T_{SH} of biomass is reached within a habitat patch, this habitat patch serves as a source habitat for emigration in the subsequent time steps and population-growth is simulated.

To calculate the population size $N_t^{\text{gro}}(v)$ per source habitat patch in each simulated time step t, the classic logistic growth function (Verhulst model[10]) is used:

$$N_t^{\text{gro}}(v) = \frac{N_{t-1}(v)K(v)}{N_{t-1}(v) + (K(v) - N_{t-1}(v))e^{-rt}},$$

where r is the intrinsic growth rate and N_{t-1} the population size at time step $t-1$. The intrinsic growth rate r was set to 2, as K.-J. Contze (personal communication, June 26, 2017) stated that habitats are fully colonized by damselflies about two to three years after an initial colonisation.

The total amount $S_{DIS} \cdot N_t(s)$ of the emitted biomass from a source habitat s is distributed to connected habitat patches. The specific share $p(v)$ that a habitat patch v receives is calculated by the following formula:

$$p(v) = \frac{C_{red}^3(v)}{\sum_{v \in Nb} C_{red}^3(v)},$$

where Nb represents the set of all not fully colonised habitat patches connected to the specific source habitat s. The reduced costs $C_{red}(v)$ ensure that close habitat patches receive a bigger share of biomass and are calculated as follows:

$$C_{red}(v) = CO_{\max} - C(v) + CO_{\min},$$

where $C(v)$ is the cost to reach an eligible habitat patch connected to the specific source habitat, $CO_{\max} = \max\{C(v) | v \in \text{Nb}\}$ is the maximum cost and $CO_{\min} = \min\{C(v) | v \in \text{Nb}\}$ is the minimum cost of all not fully colonised habitat patches connected to the source habitat. Accordingly, cost-effective connected habitat patches are preferably colonised and a value of $p(v) > 0$ consequently causes a colonisation. As soon as one of the connected habitat patches is fully colonised ($N_t(v) = K(v)$), it will be removed from Nb in the following time step until the population size in the specific patch decreases due to dispersal losses.

The colonisation success from a source habitat to an eligible habitat patch is directly related to the total value of the specific dispersal costs per connection. We assume that the specific share $p_m(v)$ of emitted biomass from a source habitat to a habitat patch decreases with increasing costs. For the calculation of $p_m(v)$ we used the following formula:

$$p_m(v) = 1 - \frac{C(v)}{C_{\max}},$$

where $C(v)$ are the costs to reach habitat patch v and $C_{\max}$ corresponds to the maximum connectivity-costs. To sum it up, the amount of incoming biomass sent to a habitat patch increases with decreasing dispersal costs.

A recalculation of the population size $N_t(v)$ for each affected habitat patch is necessary due to the previously described dispersal processes and the resulting changes in population size. At the end of every time step t, the amount of biomass $N_t^{gro}(v)$ after population growth per habitat patch is adjusted by the amount of incoming biomass $N_t^{in}(v)$ from connected source habitats. In case the specific habitat patch is a source habitat, it is furthermore necessary to subtract the emitted amount of biomass $N_t^{out}(v)$ caused by the dispersal process

$$N_t(v) = N_t^{gro}(v) + N_t^{in}(v) - N_t^{out}(v).$$

Initialisation

The simulation model was instantiated with the model parameters shown in Tab. 2. The parameter values were chosen to represent the characteristics of a European damselfly (*Coenagrion mercuriale*) where possible. To compare the optimisation model with the underlying simulation, the same parameters were used for the optimisation model. The number of time steps was adjusted, however, as a much smaller time horizon was needed for the optimisation model.

Here, we focus on developing a novel optimisation apporach. Thus, creating a realistic ecological model — and consequently choosing adequat model parameters — is only of secondary interest and we will omit a detailed discussion of the chosen parameters.

Table 2. Model parameters

PARAMETER	SYMBOL	VALUE
habitat quality	HQ	0.25-1.0
intrinsic growth rate	r	2.0
maximum connectivity-cost	$C_{\max}$	1250.0
share initial source habitats	S_{SH_i}	10.0%
share habitat patches	S_{HP}	10.0%
share biomass for dispersal	S_{DIS}	25.0%
threshold source habitat	T_{SH}	20.0
time steps	T	250

References

1. Etherington, T. R., Holland, E. P. & O'Sullivan, D. Nlmpy: a python software package for the creation of neutral landscape models within a general numerical framework. *Methods Ecol. Evol.* **6**, 164–168 (2015).
2. GeoPortal Rheinland-Pfalz. Gewässernetz (gesamt) (2017). URL http://www.geoportal.rlp.de/mapbender/php/mod_showMetadata.php?languageCode=de&resource=layer&layout=tabs&id=54712.
3. GDAL Development Team. *GDAL - Geospatial Data Abstraction Library, Version 2.2.1*. Open Source Geospatial Foundation (2017).
4. Van den Brink, P. J., Baird, D. J., Baveco, H. J. & Focks, A. The use of traits-based approaches and eco (toxico) logical models to advance the ecological risk assessment framework for chemicals. *Integr. environmental assessment management* **9** (2013).
5. Sutherland, W. J. & Norris, K. Behavioural models of population growth rates: implications for conservation and prediction. *Philos. Transactions Royal Soc. Lond. B: Biol. Sci.* **357**, 1273–1284 (2002).
6. Hepenstrick, D., Holderegger, R. & Keller, D. Monitoring von Populationen der Helm-Azurjungfer Coenagrion mercuriale (Odonata : Coenagrionidae): Was taugen zwei Begehungen pro Saison ? (2012).
7. Kastner, F., Buchwald, R. & Willen, M. Artenhilfsprogramme für die FFH- Libellenarten Aeshna viridis, Coenagrion mercuriale und Coenagrion ornatum in NW-Deutschland (2015).
8. Van der Walt, S. *et al.* scikit-image: image processing in python. *PeerJ* **2**, e453 (2014).
9. Adriaensen, F. *et al.* The application of 'least-cost'modelling as a functional landscape model. *Landsc. urban planning* **64**, 233–247 (2003).
10. Tsoularis, A. & Wallace, J. Analysis of logistic growth models. *Math. biosciences* **179**, 21–55 (2002).
